# The Seasonality of Retinal Detachment: Peaks, Troughs, and Global Trends

**DOI:** 10.3390/life15020190

**Published:** 2025-01-27

**Authors:** Georgios N. Tsiropoulos, Efstratia Amaxilati, Marianna Tranou, Eleni P. Papadopoulou, Iordanis Vagiakis, Fotis Topouzis, Georgios D. Panos

**Affiliations:** 1 First Department of Ophthalmology, AHEPA University Hospital, School of Medicine, Aristotle University of Thessaloniki, Kiriakidi 1, 54636 Thessaloniki, Greece; gntsirop@gmail.com (G.N.T.); efstratia_a@windowslive.com (E.A.); elna.pap@gmail.com (E.P.P.); jvag_@outlook.com (I.V.); ftop@auth.gr (F.T.); 2 Ophthalmica Eye Institute, 54622 Thessaloniki, Greece; marriatranoy@gmail.com; 3 Division of Ophthalmology and Visual Sciences, School of Medicine, University of Nottingham, Nottingham NG7 2UH, UK

**Keywords:** seasonality, retinal detachment, rhegmatogenous retinal detachment, meteorological factors, environmental influences, temperature, light exposure, behavioural factors

## Abstract

**Purpose:** To examine the seasonality of retinal detachment (RD) and explore global patterns and contributing factors through a narrative review. **Methods:** Studies investigating seasonal trends in RD incidence across diverse regions were analysed for peak seasons, meteorological influences, and behavioural factors. **Results:** RD seasonality varies by region. Northern climates (e.g., Finland) report summer peaks linked to prolonged daylight, while warmer climates (e.g., Kuwait) show winter peaks associated with outdoor activity. Some studies found correlations with temperature, light, and atmospheric pressure, while others reported no seasonal variation. **Conclusions:** RD seasonality reflects a complex interplay of environmental and behavioural factors. Future research should focus on standardised methodologies to clarify these relationships and inform preventative strategies.

## 1. Introduction

Retinal detachment is the separation of the neurosensory retina (NSR) from the underlying retinal pigment epithelium (RPE) [1]. This separation disrupts the normal function of the retina and can lead to permanent vision loss if not treated promptly. The forces that keep these layers in close apposition are both mechanical and metabolic in nature [2]. Mechanically, the vitreous supports the retina in place, along with the intraocular pressure. Metabolically, active processes regulate fluid balance and maintain ionic gradients to preserve retinal adhesion. Disruption in any of these mechanisms can lead to the accumulation of fluid beneath the retina, resulting in retinal detachment [2].

Whether the vitreous plays a direct role in retinal adhesion is yet to be determined, although some studies suggest that the physical structure of the vitreous might be of importance in maintaining retinal apposition [3,4].

There are four major types of retinal detachment: RRD, tractional retinal detachment (TRD), exudative or serous retinal detachment (ERD or SRD), and combined tractional rhegmatogenous retinal detachment. RRD, the most prevalent type, occurs when a retinal tear or hole allows vitreous fluid and/or misdirected aqueous to seep underneath the retina, separating it from the RPE. The degeneration of the vitreous body with age, leading to posterior vitreous detachment (PVD), is one of the main drivers of rhegmatogenous retinal detachment (RRD), the most common form of RD [1]. The main risk factors for RRD are retinal tears, myopia, and previous cataract surgery [5]. Other risk factors include vitreoretinal adhesions in association with PVD and local ocular conditions such as retinoschisis, previous trauma, and vitreoretinopathies, such as the Stickler syndrome [1]. TRDs occur as a consequence of various retinal pathologies but are most commonly associated with proliferative diabetic retinopathy [PDR] [6]. ERD occurs due to inflammatory, infectious, infiltrative, neoplastic, vascular, and degenerative conditions that may be associated with blood–retinal barrier breakdown [7].

Various studies have researched the role of seasonal variation in the occurrence of RRD [8,9,10,11,12,13,14,15]. However, the results are not unanimous, as some studies report RRD incidence with a summer peak and a winter trough [9,10,11,12] and others note a winter peak and a summer trough [8], and in some studies, no seasonal variation was found [13,14,15]. The differing results across studies emphasise the importance of exploring a range of factors—environmental, biological, and behavioural—that might influence the seasonal patterns seen in retinal detachment cases. Factors like temperature, light exposure, and atmospheric pressure have been suggested as possible contributors, with temperature changes potentially affecting the liquefaction of the vitreous and the development of PVD [16]. However, the precise mechanisms behind these seasonal variations remain unclear, and further research is needed to better understand their role.

Given these uncertainties, this review seeks to provide a comprehensive overview of the current literature on the seasonality of retinal detachment. By examining studies from various regions and climates, we aim to uncover the potential influence of geographic and weather-related factors on RD incidence. Our goal is to highlight the complex interaction of these elements and offer insights that can guide future research and clinical practice.

## 2. Methods

### 2.1. Search Strategy

We carried out a thorough and structured search of the literature to find studies that explored the seasonal variation in retinal detachment (RD). To ensure we captured a wide range of relevant studies, we used a mix of free-text keywords and Medical Subject Headings (MeSH) terms. The specific terms we focused on included “*seasonality*”, “*seasonal variations*”, “*retinal detachment*”, and “*RD*”. This approach allowed us to identify studies looking at both direct and indirect links between environmental factors and RD incidence.

We did not limit our search by publication date, ensuring we gathered as many studies as possible across different time periods. Additionally, we included articles in any language, as long as they had an English, French, or German abstract or translation available. The search strategy was refined multiple times to make sure we included all relevant studies while minimising irrelevant results.

### 2.2. Inclusion and Exclusion Criteria

#### 2.2.1. Inclusion Criteria:

Studies in which the population of interest were patients with retinal detachment.

Retrospective or prospective studies.

Studies that discussed the seasonality of the retinal detachments included in the respective study.

#### 2.2.2. Exclusion Criteria:

Studies without accessible full-text manuscripts or abstracts.

Case reports, editorials, and review articles were excluded, though relevant reviews were used to identify additional primary studies.

### 2.3. Study Selection

After the initial search, 2379 studies were identified. Titles and abstracts were screened by two independent reviewers to assess their relevance. Disagreements between reviewers were resolved through discussion, and a third reviewer was consulted when necessary.

### 2.4. Data Extraction

Data were extracted from each included study, focusing on the sample size, the study design, and the seasonal variation (if any) in the retinal detachments (peaks and troughs).

## 3. Seasonal Trends of Retinal Detachment by Region

### 3.1. Northern Europe and Siberia

Tornquist, Stenkula, and Tornquist [13], from Sweden, conducted a 10-year retrospective study that included 590 RRD cases and reported no seasonal variation in the incidence of RRD.

Laatikainen, Tolppanen, and Harju [10], from Finland, conducted a retrospective study of 310 eyes of 301 patients during a four-year period and concluded that there was a significant seasonal variation in the incidence of RRD, with a summer peak and a winter trough, attributed to prolonged daylight exposure (x^2^ = 12.98, *p* < 0.005). Paavola, Chehova, and Forsius [11] in their 5-year retrospective study that included 256 and 207 idiopathic RD cases from the Novosibirsk and Oulu regions, respectively, found out a statistically significant seasonal variation the incidence of RD in both. The highest incidence peak was found in July in Novosibirsk and in June in Oulu.

### 3.2. Central Europe

Thelen, Gerding, and Clemens [12], from Germany, conducted a retrospective study of 2314 RRD cases within a 11-year period and reported a statistically significant seasonal variation in the incidence of RRD, with a summer peak and a winter trough (*p* < 0.005). Bertelmann et al., also from Germany, conducted a 10-year retrospective study of 1490 cases and reported a statistically significant variation in the incidence of RRD, with a peak in July and a trough in October (*p* = 0.008) [17].

Weekers [18], from Belgium, performed a 15-year retrospective study that included 208 cases of idiopathic RD and presented that 31%, 30%, 21%, and 18% of the RD cases were reported in the summer, spring, autumn, and winter, respectively.

Barioulet et al. [19] conducted a 9-year retrospective study in metropolitan France (the METEO-POC study) that included 21,166 patients with idiopathic RRD. This study studied some meteorological parameters in order to understand which factors are attributed to the seasonal variations in RRD. The studied factors were the mean temperature over the preceding 10-day period from the diagnosis of the RRD (T-1), mean daily temperature over the 10 days prior T-1, minimum/maximum temperatures, rainfall, duration of sunshine, atmospheric pressure, overall radiation, relative humidity, and wind speed. Overall, they found no relationships between meteorological parameters and RRD occurrence [19].

In a nationwide database 6-year study from France conducted by Ben Ghezala et al. [20], the annual and monthly hospital incidence rates of RRD per 100,000 population were calculated for the whole country and for each region. The average monthly national RRD hospital incidence rate was the highest in June (2.03 ± 0.12 per 100,000 population) and the lowest in August (1.60 ± 0.09). The average annual age-standardised and sex-standardised regional hospital incidence rate was the highest in Guadeloupe and Pays de la Loire (28.30 ± 2.74 and 26.13 ± 0.84 per 100,000 population, respectively), and the lowest was in French Guiana and Martinique (15.51 ± 3.50 and 17.29 ± 2.12 per 100,000 population, respectively) [20].

### 3.3. South Europe and Mediterranean

Ivanisevic et al., from Croatia, conducted a 12-year retrospective review of medical records, including 280 eyes of 272 patients with RRD, and they reported no seasonal variations in the occurrence of RRD [15].

Sevillano Torrado et al. [21], from Spain, performed a 6-year retrospective study of 256 RRD cases and reported a statistically significant seasonal variation in the incidence of RRD. The maximum number of incident cases was observed in June and July, and the minimum number of cases was observed in January and December [21].

In a 9-year retrospective study of 363 patients from Italy with RRD, Ghisolfi, Vandelli, and Marcoli [9] reported a significant seasonal variation in the incidence of RRD, with a peak in the summer months. They performed a trimestrial cumulative RRD case number categorisation and found that most RRDs occurred in the trimesters April–May–June and July–August–September [9].

Erdol et al. [22], from Turkey, performed an 8-year retrospective study of 286 RRD cases from 276 patients and did not determine a statistically significant monthly variation (*p* = 0.613).

### 3.4. United States

In a 3-year retrospective, cross-sectional, population-based study of 12,492 RRD cases, Shaheen et al. concluded that there was no statistically significant seasonal variation in the incidence of RRD (*p* = 0.819) [23].

### 3.5. China and Asia

In a 1-year prospective population-based incidence study from China, no seasonal variation was reported in 478 RRD cases. No seasonal variation was also reported when the RRD cases were divided into subtypes of RRD (blunt traumatic, aphakic and pseudophakic, nontraumatic phakic) [24].

Zou, Zhang, Xu, Wang, Liu, and Ho [14] reported no seasonal variation in 61 RRD cases that occurred between January 1996 and December 1999 in Beixinjing District, Shanghai.

In a 2-year retrospective review of 76 patients with RRD, Prabhu and Raju [25], from India, reported that RRD showed a definite seasonal variation that peaks in the summer months.

Iida et al. [26], from Japan, conducted a 5-year retrospective ecological study of 571 eyes of 543 patients with RRD and reported no seasonal variation in the incidence of RRD (*p* = 0.77).

In a retrospective chart review of 211 patients from Lebanon with idiopathic RRD, Mansour et al. [27] reported a significant seasonal variation (*p* < 0.05) with an increase in RRD in the warm seasons (spring and summer) compared to the cold seasons (winter and autumn; 56 vs. 44%) in one referral medical centre over a 13-year period.

Lin et al. [28], from Taiwan, conducted an 11-year nationwide population-based study of 23,718 eyes with RD, which showed a significant seasonal variation in the incidence of RD. The authors found that the highest rates occurred between August through October, which strongly correlated with higher ambient temperatures and lower atmospheric pressures (*p* < 0.05); they decreased in November and were lowest in February [28].

In a 6-year retrospective study of 266 eyes of 259 patients with RD from Kuwait, Al Samarrai [8] reported a seasonal variation in the incidence of RD, with a peak in the winter months and a trough in the summer months (*p* = 0.0453).

### 3.6. Australia

Manners et al. [29] conducted a 13-year whole-population retrospective observational study that included 4376 eyes with RD and reported no seasonal variation in the incidence of RD.

A summary of the studies is depicted in Table 1, while a concise overview of the seasonal trends in RRD incidence based on geographic regions, reported seasonal peaks, and associated meteorological factors is outlined in Table 2.

## 4. Discussion and Synthesis of Findings

Retinal detachment is a serious retinal condition globally that can lead to blindness if left untreated. Prompt identification of the condition greatly enhances the likelihood of a successful repair and vision improvement [30]. The annual incidence of RD is approximately 7–14 per 100,000 cases [22,31], though this rate varies greatly across different geographic locations, ethnicities, age groups, and patient anatomical characteristics such as myopia [16]. Additionally, a seasonal variation in RD incidence has been proposed by many authors in recent years. To indicate the scale of seasonal variation, we provide the following quantitative data: Laatikainen et al. [10] reported a significant seasonal variation in Finland with the incidence of RD peaking in the summer months, specifically in June and July. The variation was statistically significant (x^2^ = 12.98, *p* < 0.005). Bertelmann et al. [17], in Germany, observed peaks in July and troughs in October, with a statistical significance of *p* = 0.008. In Belgium, Weekers [18] identified that 31% of cases occurred in summer, 30% in spring, 21% in autumn, and 18% in winter. Conversely, Al Samarrai et al. [8] reported the highest incidence in Kuwait during the coldest winter months (*p* = 0.0453).

Finnish summers and Kuwaiti winters present stark contrasts in climate and environmental conditions, yet both show seasonal peaks in RD incidence. Finnish summers feature prolonged daylight hours and higher temperatures, which may accelerate vitreous liquefaction and posterior vitreous detachment (PVD). Conversely, Kuwaiti winters, characterised by cooler temperatures (Kuwait experiences milder and more pleasant weather during the winter months compared to extremely high summer temperatures), might see increased outdoor activities, leading to trauma-induced RD. These findings suggest that environmental and behavioural factors interact uniquely in different climates, emphasising the multifactorial nature of RD seasonality.

All of the reviewed studies, which were conducted in different regions and time periods, attempted to identify any significant differences in the frequency of retinal detachments across the seasons. However, the reported results highlight considerable variability, reflecting the complex interplay of environmental, biological, and behavioural factors. More specifically, a higher incidence of RD during warmer seasons was noted in studies conducted in France [20], Asia [25], Finland [10,11], Germany [12,17], Spain [21], Lebanon [27], Taiwan [28], Belgium [18], and Italy [9]. On the other hand, a study from Kuwait observed a peak incidence of RD during the coldest winter months [8]. There were also studies that failed to reveal a significant difference between the seasons from Australia [29], China [14,24], Turkey [22], Croatia [15], Sweden [13], Japan [26], and the United States [23]. While summer peaks dominate in northern climates, there is a report by Al Samarrai et al. [8] that observed a higher incidence of RD in the colder winter months in Kuwait. Although it is the result of only one study, we believe that warmer regions may have a higher RRD incidence in winter, possibly due to seasonal shifts in outdoor activities and temperature tolerance.

Furthermore, eleven of the aforementioned studies, in order to thoroughly examine the seasonal variation in retinal detachments, included in their evaluation specific meteorological factors like temperature, humidity, precipitation, atmospheric pressure, and sunlight exposure. Again, the results of the studies were contradictory regarding the effect of environmental factors on the incidence of RD. No association was indicated between the meteorological factors and the frequency of RD in four of the studies [15,17,19,27], whereas in some studies, a positive correlation was noted with increased temperature [25,28], greater sunlight exposure [9,12,21,25], and humidity [25]. Oppositely, Al Sammarai et al. observed a negative correlation with temperature and average sunlight exposure [8]. Lastly, atmospheric pressure was inversely associated with RD incidence by Lin et al. [28] and Lida et al. [26]. Hence, the above studies have suggested that environmental factors may contribute to the onset of RD, possibly explaining its higher incidence during certain months. Nevertheless, the results were inconclusive due to their conflicting nature.

Several mechanisms involving environmental factors have been proposed to justify the seasonal variation in the occurrence of retinal detachments; however, their confirmation is still awaited. It is widely accepted, however, that events such as liquefaction, traction, and detachment of the vitreous and intraocular fluid movement, along with deterioration of retinal adhesion, play key roles in RD [1,32]. Retinal adhesion, defined as the apposition of the neurosensory retina to the RPE, is maintained through mechanical and metabolic forces. Mechanical forces include oncotic pressures, properties of interphotoreceptor matrix, and interdigitations of RPE, while metabolic forces are mainly processed through oxygenation via the RPE pump and the release of lysosomal enzymes [1]. These physiological properties can be disrupted by environmental factors [16].

The most frequently examined meteorological factors influencing the onset of RD are elevated temperature and extreme exposure to UV light [28]. First of all, higher temperatures are thought to cause retinal damage by affecting the vitreous viscosity, leading to posterior vitreous detachment [33,34]. Specifically, it has been suggested that increased temperatures cause dehydration, leading to the disruption of osmotic pressures between the blood circulation and the tissues of the eye. This may alter the volume of the vitreous, causing shrinkage and ultimately detachment. However, the occurrence of acute vitreous detachment through this process has not been fully elucidated [25]. In contrast, lower temperatures have been proven in animal models to strengthen the adhesion of the retina [35,36].

Moreover, UV light has been found to cause posterior vitreous detachment through vitreous liquefaction due to free radical action and destabilisation of glycosaminoglycans in the interphotoreceptor matrix [25,37,38]. Daylight and temperature are also known to affect cortisol levels in the blood circulation and retina. Hence, during summer months, the concentration of cortisol in the retina decreases [39,40]. Low subretinal cortisol levels have been implicated to some extent in the destabilisation of retinal adhesion by disrupting the glycosaminoglycan matrix and inhibiting the blockage of RPE lysosomal enzymes [9,10].

As mentioned earlier, vitreoretinal traction is a well-known risk factor for RRD [1]. Therefore, conditions that may increase vitreous movement and subsequent traction are believed to contribute to the occurrence of retinal detachment. These conditions include excessive eye rubbing related to ocular surface disease and seasonal allergies [27,41,42], which have been observed to increase during the spring and summer months, as well as excessive physical activity [11]. In a review, Kriebel et al. highlight the part hard lifting plays in the development of RD [43]. In a case–control study, Mattioli et al. included 99 myopic controls and 61 cases of RD and myopia [44]. According to the study, hard lifting was associated with a higher chance of developing RD (odds ratio = 4.4 [95% CI = 1.5–13] compared to no lifting). Furthermore, Farioli et al. concluded that heavy occupational lifting is a risk factor for RRD based on data from a Swedish cohort of 43,321 males [45].

A summary of all the possible mechanisms is given below. Elevated temperatures have been proposed to increase vitreous liquefaction, potentially accelerating posterior vitreous detachment (PVD). This process involves dehydration and disruption of osmotic pressures, altering vitreous volume and increasing traction forces on the retina [25,33,34]. High UV light exposure may lead to vitreous destabilisation through free radical damage to glycosaminoglycans, contributing to retinal detachment [25,37]. Seasonal variations in cortisol levels, influenced by temperature and daylight, may weaken retinal adhesion by disrupting the glycosaminoglycan matrix and interfering with the regulation of lysosomal enzymes [9,10,39,40]. Low atmospheric pressure, observed in some studies to correlate with higher RD rates [26,28], might impact the dynamics of intraocular fluid movement and vitreoretinal adhesion.

All these mechanisms seem to play a cumulative role in the occurrence of retinal detachments, possibly ultimately influencing their seasonal variation.

It should be noted that seasonal trends exhibit significant fluctuation depending on the region. Examining these trends in a variety of geographical areas reveals clear seasonal peaks in the incidence of RD, especially in areas like France [20], Finland [10,11], and Spain [21], where the incidence of RD cases is lower in the winter and significantly higher in the summer. Variations in temperature, light exposure, and atmospheric pressure may contribute to the occurrence of RD, as these regional patterns may be impacted by local environmental and lifestyle factors.

For example, variations in temperature have been proposed as possible causes of RD seasonality. In their evaluation of a number of meteorological factors, Barioulet et al. discovered that high temperatures may hasten the vitreous humour’s liquefaction, thereby raising the risk of PVD during warmer months H [19]. The vitreoretinal interface may also be impacted by variations in atmospheric pressure, as some studies have found a relationship between higher RD rates and lower atmospheric pressures [16,19]. These results raise the possibility that weather and climate may physiologically affect vitreous dynamics and structure, which calls for more research. Another impact could be the amount of sunlight, which fluctuates greatly depending on the season.

In regions with higher seasonal variability in daylight hours, increased exposure to sunlight during the summer may correspond to a higher rate of outdoor activities, which could elevate the risk of minor ocular trauma or increase visual demands, both of which may contribute to RD development [16,21].

Moreover, aspects connected to behaviour and activity might be significant. Higher levels of physical activity are frequently associated with regions where the prevalence of RD rises during warmer months. In these situations, a higher risk of retinal tears or mild traumas that could predispose people to RD could be attributed to an increase in outdoor sports and activities. The prevalence of RD in these areas lends credence to the idea that seasonal changes in lifestyle can raise the risk of RD, particularly when paired with environmental factors like more sunshine and warmer temperatures. However, a number of studies conducted in places like Sweden and Japan show no significant seasonal change in the prevalence of RD, indicating that regional variations in environment, lifestyle, or even genetics may lessen or obscure seasonal effects [13,26]. This lack of seasonal variability highlights the complex interaction of behavioural and environmental factors in RD seasonality and suggests that the influence of weather may not be universally applicable.

Seasonal bias due to healthcare system accessibility and reporting practices may also contribute to the observed variation in RD incidence. Regions with fluctuating healthcare availability—either from seasonal closures, staff shortages, or access issues during extreme weather—may report fewer cases during these periods. For instance, individuals in rural or underserved areas may delay seeking care during winter months, potentially resulting in a lower reported incidence of RD that does not reflect actual occurrence rates. Additionally, reporting biases can arise if certain providers more rigorously record cases at specific times of year, such as following holiday periods. This could create the appearance of seasonal trends where none exist. Additionally, research from nations with consistent and universal access to healthcare, like Sweden and Japan, indicates that there is no discernible seasonal variation in the incidence of RD, indicating that reported seasonal trends may be influenced by healthcare accessibility [13,26]. These factors emphasise how crucial it is to take the healthcare system and reporting biases into account when analysing seasonal patterns in RD, since they have the potential to mask or overstate actual seasonal impacts.

Many studies fail to standardise their methodologies, including variations in defining seasonal periods and meteorological factors. This complicates cross-regional comparisons and underscores the need for multicentre studies with unified protocols to clarify the role of environmental influences on RD. Additionally, inconsistent reporting practices and varying healthcare access between regions may contribute to the conflicting findings. Addressing these methodological challenges will be critical for future research to provide clearer insights into the seasonal variation in RD. It should also be stated that although numerous studies have investigated the relationship between biological and meteorological factors and the incidence of retinal detachment, none of these factors have been definitively proven to cause seasonal variations or an overall increase in RD cases. The associations observed in the reviewed studies remain correlational, with mechanisms largely hypothesised rather than empirically validated.

Future research on the seasonality of retinal detachment (RD) should aim to address key limitations in the current literature, focusing on enhancing both the geographic scope and methodological rigour of studies. To comprehensively understand how specific environmental factors—such as temperature fluctuations, atmospheric pressure changes, and variations in sunlight exposure—may influence RD incidence, longitudinal, multiregional studies are warranted. Such studies, spanning diverse climates and healthcare systems, would facilitate a more granular analysis of seasonal patterns and environmental determinants across populations. In addition, there is a need for controlled investigations that isolate individual meteorological factors, employing both experimental and observational methods, to delineate the mechanisms through which these variables may affect RD incidence. This approach would enable a clearer understanding of the precise impact of each factor, which remains ambiguous in current research. The role of behavioural patterns and physical activity levels, particularly in warmer months associated with increased RD incidence, also requires further exploration. Studies that correlate specific behavioural data—such as participation in outdoor activities, screen exposure, and minor ocular traumas—with RD incidence could provide critical insights into how lifestyle factors contribute to seasonal variations in RD risk. Healthcare system accessibility and reporting practices are additional variables that may introduce seasonal bias in RD data, and future studies should incorporate these factors as covariates, especially in rural or seasonally isolated regions. Investigating how healthcare availability and variability in reporting influence the perceived seasonality of RD would yield a more accurate understanding of its true epidemiology.

Moreover, examining genetic and ethnic factors may elucidate geographical differences in RD incidence, as genetic predispositions influencing vitreoretinal health could modulate the impact of seasonal environmental exposures. Multigenerational studies or population-specific cohort analyses could provide valuable data on potential genetic contributions to seasonal trends in RD.

Finally, the development of predictive models for RD risk, based on seasonal patterns and associated behavioural and environmental variables, would be beneficial. Such models could support healthcare systems in pre-emptively identifying periods of increased RD risk, thereby facilitating timely preventative measures and optimising clinical resource allocation. These proposed research directions underscore the need for a multidimensional approach to unravel the complexities of RD seasonality.

Although findings vary across regions and studies, this review provides a comprehensive synthesis of the seasonal trends in RD. By consolidating diverse evidence, it highlights the need for standardised methodologies to reveal the complex interplay between environmental, biological, and behavioural factors. Such efforts will inform preventative strategies and optimise clinical outcomes.

## Figures and Tables

**Table 1:** Summary of the studies on RD seasonality.

Study	Design	Country	Number of Patients with Rd	Key Findings
Törnquist et al. (1987) [13]	10-year retrospective study	Sweden	590 RRD cases	No seasonal variation in the incidence of RRD
Laatikainen et al. (1985) [10]	4-year retrospective study	Finland	301 patients (310 eyes with RRD)	Statistically significant seasonal variation in the RRD incidence Summer peak/winter trough
Paavola et al. (1983) [11]	5-year retrospective study	Finland	256 idiopathic RRD patients (Novosibirsk) 207 idiopathic RRD patients (Oulu)	Statistically significant seasonal variation in the incidence of RRD Summer peak in July (Novosibirsk) and June (Oulu)/winter trough
Thelen et al. (1997) [12]	11-year retrospective study	Germany	2314 patients	Statistically significant seasonal variation in the occurrence of RRD Mid-summer peak (July *n* = 228)/winter trough (December–January *n* = 161) Close relation between the RRD seasonal variation and the seasonal changes in daylight duration
Bertelmann et al. (1997) [17]	10-year retrospective study	Germany	1490 cases	Statistically significant seasonal variation in RRD incidence July peak/October trough No significant impact of sunlight and temperatures on RRD 1.43% fewer RRDs during school holidays
Weekers (1945) [18]	15-year retrospective study	Belgium	208 cases of idiopathic RD	31% cases in the summer/18% in the winter
Barioulet et al. (2024) [19]	9-year retrospective study	France	21,166 patients of idiopathic RRD	No relationship between meteorological parameters (min/max temperatures, sunshine durations, atmospheric pressure, overall radiation, relative humidity, wind speed) and RRD occurrence
Ben Ghezala et al. (2022) [20]	6-year national database study	France	Annual and monthly hospital incidence rates of RRD per 100,000 population measured for the whole country and each region	June peak/August trough The hospital incidence rate of RRD varied by season and geographic location
Sevillano Torrado et al. (2020) [21]	6-year retrospective study	Spain	256 RRD cases	Significant seasonal variation in the incidence of RRD June/July peak/January/December trough A positive correlation was found between RRD incidence and solar radiation
Shaheen et al. (2023) [23]	3-year retrospective, cross-sectional, population-based study	United States	12,492 RRD cases (mostly 50–64 years)	No significant seasonal variation in RRD incidence
Ghisolfi et al. (1986) [9]	9-year retrospective study	Italy	363 patients	Significant seasonal variation in the incidence of RRD Positive correlation between RRD incidence and light flux values (light may precipitate RRD in a damaged retina) Summer peak
Erdol et al. (2020) [22]	8-year retrospective study	Turkey	286 RRD cases	No significant seasonal variation in the incidence of RRD
Li (2003) [24]	1-year prospective population-based incidence study	China	478 RRD cases	No significant seasonal variation in the incidence of RRD
Zou et al. (2002) [14]	3-year study	China	61 RRD cases	No significant seasonal variation in the incidence of RRD
Prabhu and Raju (2016) [25]	2-year retrospective review	Asia	76 RRD cases	Significant seasonal variation in the incidence of RRD Summer peak/winter trough Meteorological factors (peak temperatures, sun hours, high humidity, low rainfall) potentially influencing the onset of RRD
Lida et al. (2021) [26]	5-year retrospective ecological study	Japan	543 patients (571 eyes with RRD)	No significant seasonal variation in the incidence of RRD
Mansour et al. (2009) [27]	13-year retrospective chart review	Lebanon	211 patients with idiopathic RRD	Significant seasonal variation in the incidence of RRD Warm seasons (spring, summer) peak Right eyes were affected more than left Onset age is younger in warm vs. cold seasons.
Lin et al. (2011) [28]	11-year nationwide population-based study	Taiwan	23,718 eyes with RRD	Significant seasonal variation in the incidence of RRD August–October peak/February trough Monthly RD incidence was positively associated with temperature and negatively with atmospheric pressure
Al Samarrai (1990) [8]	6-year retrospective study	Kuwait	259 patients (266 eyes with RD)	Significant seasonal variation in the incidence of RD Winter peak (November)/summer trough
Manners et al. (2017) [29]	13-year whole-population retrospective observational study	Australia	4376 eyes with RD	No seasonal variation in the incidence of RD
Ivanisević et al. (2002) [15]	12-year retrospective review of medical records	Croatia	280 eyes of 272 eyes with RRD	No seasonal variations in the occurrence of RRD.

**Table 2:** A concise overview of the seasonal trends in RRD incidence based on geographic regions, reported seasonal peaks, key findings from studies, and associated meteorological factors.

Region	Seasonal Peak	Key Findings	Associated Meteorological Factors
**Finland (Helsinki)**	Summer (July, August)	Significant summer peaks linked to prolonged daylight.	Prolonged daylight, increased light exposure.
**Germany** **(Münster)**	Summer (July)	36% higher incidence in summer; strong correlation with daylight hours.	Astronomic daylight duration, extended light exposure.
**Taiwan**	Late summer/fall (August–October)	Positive correlation with higher ambient temperature and lower atmospheric pressure.	High temperature, low atmospheric pressure.
**Kuwait**	Winter	Increased cases during cooler months, potentially due to elevated outdoor activities.	Cooler temperatures encouraging outdoor activities.
**Metropolitan France**	Winter (mild months)	Weak correlation between RRD and meteorological factors; slight increase in incidence in winter.	Minimal meteorological correlation identified.
**Western Australia**	None	Stable annual incidence; no significant seasonal variation.	No specific association reported.
**United States (IRIS Registry)**	None	Minimal seasonal variation; changes linked to holidays rather than specific seasons.	National holidays impact, not meteorological factors.

## Data Availability

Not applicable.
